# Association of non-highdensity lipoprotein cholesterol to highdensity lipoprotein cholesterol ratio (NHHR) and subsequent hypertension and heart diseases: findings from the CHARLS cohort

**DOI:** 10.1007/s40520-024-02919-z

**Published:** 2025-01-21

**Authors:** Feng Zhang, Zhuqing Li, Meng Wang, Yanxin Wang, Chengzhi Lu

**Affiliations:** 1 The First Central Clinical School, Tianjin Medical University, Tianjin, 300070 China; 2 Department of Cardiology, Tianjin First Center Hospital, Tianjin, 300192 China

**Keywords:** Hypertension, Heart diseases, Non-HDL-c, NHHR, Nonlinear relationship, CHARLS

## Abstract

**Purpose:**

NHHR, the ratio of non-high-density lipoprotein cholesterol to high-density lipoprotein cholesterol, is a novel lipid marker associated with the risk of heart diseases and various health conditions. However, there is limited evidence regarding the relationship between NHHR and the onset of hypertension and heart diseases. The aim of this study was to investigate the association between NHHR and the new-onset hypertension and heart diseases among the Chinese middle-aged and older general population.

**Methods:**

This prospective cohort study utilized data from a nationally representative sample of Chinese residents aged 45 and older, sourced from the China Health and Retirement Longitudinal Study (CHARLS). The primary outcomes of the study were new-onset hypertension and heart diseases. To investigate the relationship between the NHHR and the risk of new-onset hypertension and heart diseases, multivariate logistic regression models and the restricted cubic spline (RCS) method were employed. Additionally, the mediating effects of hypertension on the association between NHHR and heart diseases were assessed using the bootstrap method.

**Results:**

A total of 5349 participants were included in the final analysis and three groups of NHHR were identified, including low-stable, medium-stable, and high-stable NHHR. By 2020, 1,631 participants (30.5%) had been newly diagnosed with hypertension, while 1233 (23.1%) developed heart diseases. Compared to those with a low-stable NHHR, individuals in the other two groups showed a significantly increased risk of developing hypertension and heart diseases. The findings remained consistent across various sensitivity analyses. According to the RCS analysis, a partial U-shaped relationship existed between the NHHR and the risk of developing hypertension and heart diseases (P for nonlinear < 0.001). Furthermore, hypertension was found to partially mediate the association between NHHR and heart diseases.

**Conclusion:**

The NHHR was closely associated with an increased risk of developing hypertension and heart diseases. In addition, the NHHR partially mediated the development of heart diseases by promoting hypertension progression. In the prevention and treatment of heart diseases, managing both lipid levels and blood pressure is crucial.

**Supplementary Information:**

The online version contains supplementary material available at 10.1007/s40520-024-02919-z.

## Introduction

Hypertension and heart diseases are significant global health concerns, which are two well-known major risks for cardiovascular mortality [1–2]. Cardiovascular diseases (CVDs) primarily affect middle-aged and elderly patients; however, recent trends indicate an increasing prevalence among younger individuals [3]. Hypertension is closely related to the development of heart diseases. Persistently elevated blood pressure damages the arteries, reducing their elasticity and increasing susceptibility to the accumulation of fatty plaques (atherosclerosis) [4]. This process can restrict blood flow, leading to conditions such as coronary heart diseases, heart attacks, strokes, heart failure, and peripheral arterial diseases. According to the World Health Organization (WHO), over 85% of all CVD-related deaths result from heart attacks and strokes, conditions that are significantly influenced by hypertension [5–6]​.

NHHR stands for the” non-high-density lipoprotein cholesterol to high-density lipoprotein cholesterol ratio” [7–8]. It is a novel lipid marker used to assess cardiovascular diseases risk. This ratio provides insight into lipid metabolism and has been associated with various health conditions, including hypertension, cardiovascular diseases, and hyperuricemia. Recent studies indicate that a higher NHHR is associated with a greater risk of these conditions, like CVDs, Diabetes mellitus, Hyperuricemia [9–12]. NHHR is considered a more comprehensive lipid indicator compared to traditional single lipid parameters, as it reflects the balance between harmful (Non-HDL-c) and protective (HDL-c) cholesterol, making it a valuable marker for evaluating cardiovascular health [13–14].

Recent studies involving the American population have shown that NHHR is positively correlated with hypertension and cardiovascular mortality [15–16]. However, related research in other populations is lacking to support these findings. Therefore, using data from the China Health and Retirement Longitudinal Study (CHARLS), we collected NHHR and related information from a general middle-aged and elderly population. Subsequently we tracked the incidence of hypertension and cardiovascular diseases over the following seven years to assess their association. In addition, we evaluated the mediating effect of new-onset hypertension on the relationship between NHHR and the incidence of cardiovascular diseases.

## Methods

### Study design and participants

The China Health and Retirement Longitudinal Study (CHARLS) is a nationally representative longitudinal survey that collects high-quality data on the health, economic, and social conditions of Chinese residents aged 45 and older. The study aims to understand the aging process and the factors affecting the well-being of older adults in China. It covers a wide range of information, including demographic characteristics, family structure, health status, healthcare utilization, employment, retirement, income, and assets. CHARLS was initiated by Peking University in 2011, modeled after the Health and Retirement Study (HRS) in the United States. The baseline survey involved nearly 17,500 individuals from 28 provinces in China, and follow-up surveys have been conducted every two to three years.

We used the 2011 population survey results as baseline data for the study, which included a total of 15,856 participants. Subsequently we tracked the incidence of hypertension and heart diseases among the participants in follow-up surveys conducted in 2013, 2015, 2018, and 2020. Patients who had heart diseases or hypertension at the baseline survey in 2011, those with missing blood test indicators, incomplete medical history, missing physical examination data, lost to follow-up, or those who died from causes other than hypertension and heart diseases, were excluded. This process resulted in a final dataset of 5,349 participants.

### Exposure and outcome identification

In this study, the NHHR (Non-HDL cholesterol to HDL-cholesterol ratio) was used as the exposure variable. Non-HDL cholesterol was calculated by subtracting HDL cholesterol from total cholesterol (TC), using blood samples collected from fasting participants [17–18]. The primary outcomes of this study were new-onset hypertension and heart diseases (including stroke) based on follow-up data collected in 2013,2015,2018,2020. New-onset hypertension was identified based on self-reported doctor diagnoses and the use of any antihypertensive medication. Similarly, new cases of heart diseases were determined through self-reported doctor diagnoses of stroke, heart attack, coronary heart diseases, angina, heart failure, or other heart-related issues, along with self-reported treatment for any of these conditions.

### Covariates

The study considered various potential covariates, including demographics (age, sex, marital status [married or others], and educational attainment [junior high school and below or high school and above]) and medical history (smoking status [never or former], alcohol consumption [never or former], diabetes mellitus, dyslipidemia, liver disease, lung disease, kidney disease, and cancer), obtained by trained interviewers using standardized questionnaires. Blood pressure and pulse were measured three times on the participants’ left arm by trained staff using an automated electronic device at 45-second intervals. The average of these three measurements was used in the study. Trained health workers measured participants’ weight and height using calibrated equipment, with participants wearing light clothing and no shoes. Body Mass Index (BMI) was then calculated as weight in kilograms divided by height in meters squared (kg/m²). Waist circumference was measured at the midpoint between the lowest rib and the iliac crest using a nonelastic tape. Trained nurses collected 8 milliliters of venous blood from participants who had fasted for at least 8 h. The samples were stored at -80 °C until analysis at the central laboratory. All covariates were collected from the baseline survey of 2011.

### Statistical analysis

Statistical analyses were conducted using IBM SPSS Statistics 26 (https://www.ibm.com/cn-zh/spss) and R software (http://www.R-project.org). A two-sided P-value < 0.05 was considered statistically significant. The participants were divided into three groups based on the tertiles (low-stable, medium-stable, and high-stable NHHR) of the NHHR. Baseline characteristics across the NHHR groups were compared using one-way ANOVA, the Kruskal–Wallis H-test, and chi-square tests, as appropriate. Continuous variables were presented as means with standard deviations (SD), while categorical variables were expressed as frequencies and percentages. Three models were established to control for confounding factors, and a weighted multivariable logistic regression model was used to estimate the relationship between NHHR and new-onset hypertension and heart disease. Model 1 did not include any covariate adjustments. Model 2 was adjusted for age, sex, marital status. Model 3 was further adjusted for education, hemoglobin, creatinine, smoke, alcohol drink, cancer, lung diseases, kidney diseases and liver diseases. The variance inflation factor (VIF) was further applied to detect multicollinearity in regression analyses. Decision curve analysis (DCA) and receiver operating characteristic (ROC) curve analysis were used to evaluate the effectiveness of Non-HDL cholesterol, HDL cholesterol, LDL cholesterol, and NHHR in predicting the risk of developing hypertension and heart disease. Restricted cubic spline (RCS) analysis was used to explore potential nonlinear relationships between NHHR and hypertension and heart diseases, adjusting for the same covariates included in the multivariable logistic models. The Akaike Information Criterion (AIC) was used to determine the optimal number of knots, selecting a value between 3 and 7 [19]. Once significant associations between NHHR groups and hypertension and heart diseases were established, the bootstrap method with 1,000 resamples was employed to assess the mediation effect of hypertension on the relationship between NHHR and cardiovascular diseases (CVDs) [20].

## Results

### Baseline characteristics

A total of 5,349 participants were included in our study, with an average age of 57.78 ± 9.48 years. Among them, 46.27% were male, 89.33% were married, and 90% had a high school education or lower. 38.18% of participants had a history of smoking, and 38.75% were regular alcohol consumers. Table 1 presents baseline characteristics of the participants according to NHHR tertiles. The most common comorbidity was lung diseases (8.62%), followed by lipid disorders (5.35%) and kidney diseases (4.67%). There were no significant differences in age, sex, or education level among participants with different NHHR levels. However, the proportion of married individuals was relatively higher among those with elevated NHHR levels. Meanwhile compared to participants in the lowest tertile, those with higher NHHR had slightly higher blood pressure, pulse rate, and waist circumference (still within the normal range). Furthermore, in terms of hematological examination data, they showed relatively higher levels of hemoglobin, TC (total cholesterol), TG (triglycerides), LDL-c (low-density cholesterol), creatinine, FBG (fasting blood glucose), and HbA1c (glycated hemoglobin), but had relatively lower levels of HDL-c (high-density cholesterol). Participants with higher NHHR levels had a higher proportion of alcohol consumption and were more likely to have lung disease, diabetes, and lipid disorders.

**Table 1:** Baseline characteristics according to NHHR tertiles

Characteristics NHHR	Overall (0.40-20.62)	Low-NHHR (< 2.21)	Medium-NHHR (2.21–3.23)	High-NHHR (> 3.23)	*P* value
	(*n* = 5349)	(*n* = 1622)	(*n* = 1941)	(*n* = 1786)	
**Demographic**					
Age (years)	57.78 ± 9.48	58.32 ± 9.90	57.62 ± 9.57	57.46 ± 8.96	0.023
Male [n (%)]	2475(46.27%)	779(48.03%)	880(45.34%)	816(45.69%)	0.230
Married [n (%)]	4778(89.33%)	1422(87.67%)	1189(89.85%)	1612(90.26%)	< 0.001
**Education attainment**					0.013
Junior high school and below [n (%)]	4814(90.00%)	1486(91.62%)	1746(89.95%)	1582(88.58%)	
High school and above [n (%)]	535(10.00%)	136(8.38%)	195(10.05%)	204(11.42%)	
**Physical examination**					
Systolic blood pressure (mmHg)	122.31 ± 16.04	120.71 ± 16.55	121.90 ± 16.09	124.22 ± 15.31	< 0.001
Diastolic blood pressure (mmHg)	72.87 ± 11.07	71.31 ± 11.28	72.62 ± 10.83	74.56 ± 10.91	< 0.001
Pulse (beat per minute)	72.01 ± 10.12	71.66 ± 10.41	71.29 ± 9.90	73.11 ± 9.99	< 0.001
Body mass index (kg/m^2^)	23.60 ± 34.56	21.63 ± 3.13	24.57 ± 57.14	24.35 ± 3.73	0.022
Waist circumference (cm)	82.79 ± 11.51	79.28 ± 9.66	82.30 ± 11.23	86.51 ± 11.51	< 0.001
**Laboratory**					
Hemoglobin (g/dl)	14.29 ± 2.21	13.97 ± 2.20	14.26 ± 2.17	14.62 ± 2.22	< 0.001
Triglyceride (mg/dl)	121.32 ± 87.15	76.24 ± 31.12	103.10 ± 45.72	182.05 ± 117.08	< 0.001
Total cholesterol (mg/dl)	192.23 ± 37.59	175.07 ± 31.22	190.62 ± 33.53	209.58 ± 39.44	< 0.001
LDL-c (mg/dl)	116.08 ± 34.02	96.16 ± 23.42	119.47 ± 27.71	130.49 ± 39.42	< 0.001
HDL-c (mg/dl)	52.53 ± 15.20	66.46 ± 14.16	52.47 ± 9.65	39.95 ± 8.59	< 0.001
Creatinine (mg/dl)	0.76 ± 0.18	0.75 ± 0.17	0.76 ± 0.17	0.77 ± 0.18	< 0.001
FBG (mg/dl)	107.12 ± 30.76	102.77 ± 24.40	104.37 ± 25.62	114.04 ± 38.83	< 0.001
Triglyceride and glucose index(mg/dl)^2^	8.59 ± 0.62	8.18 ± 0.42	8.49 ± 0.46	9.07 ± 0.62	< 0.001
HbA1c (%)	5.23 ± 0.76	5.13 ± 0.63	5.19 ± 0.65	5.37 ± 0.93	< 0.001
**Comorbidity**					
Smoked [n (%)]	2042(38.18%)	641(39.52%)	722(37.20%)	679(38.02%)	0.359
Alcohol drink [n (%)]	2073(38.75%)	700(43.16%)	732(37.71%)	641(35.89%)	< 0.001
Diabetes mellitus [n (%)]	195(3.65%)	36(2.20%)	61(3.14%)	98(5.49%)	< 0.001
Dyslipidemia [n (%)]	286(5.35%)	47(2.90%)	88(4.53%)	151(8.45%)	< 0.001
Liver diseases [n (%)]	151(2.82%)	51(3.14%)	59(3.04%)	41(2.30%)	0.252
Lung diseases [n (%)]	461(8.62%)	176(10.85%)	149(7.68%)	139(7.78%)	0.001
Kidney diseases [n (%)] Cancer [n (%)]	250(4.67%) 39(0.73%)	84(5.18%) 7(0.43%)	87(4.48%) 17(0.86%)	79(4.42%) 15(0.84%)	0.512 0.239

HDL-c, high-density cholesterol; LDL-c, low-density cholesterol; FBG, fasting blood glucose; HbA1c, glycated hemoglobin

### Association of the NHHR groups and new-onset hypertension and heart diseases

**Fig. 1:**
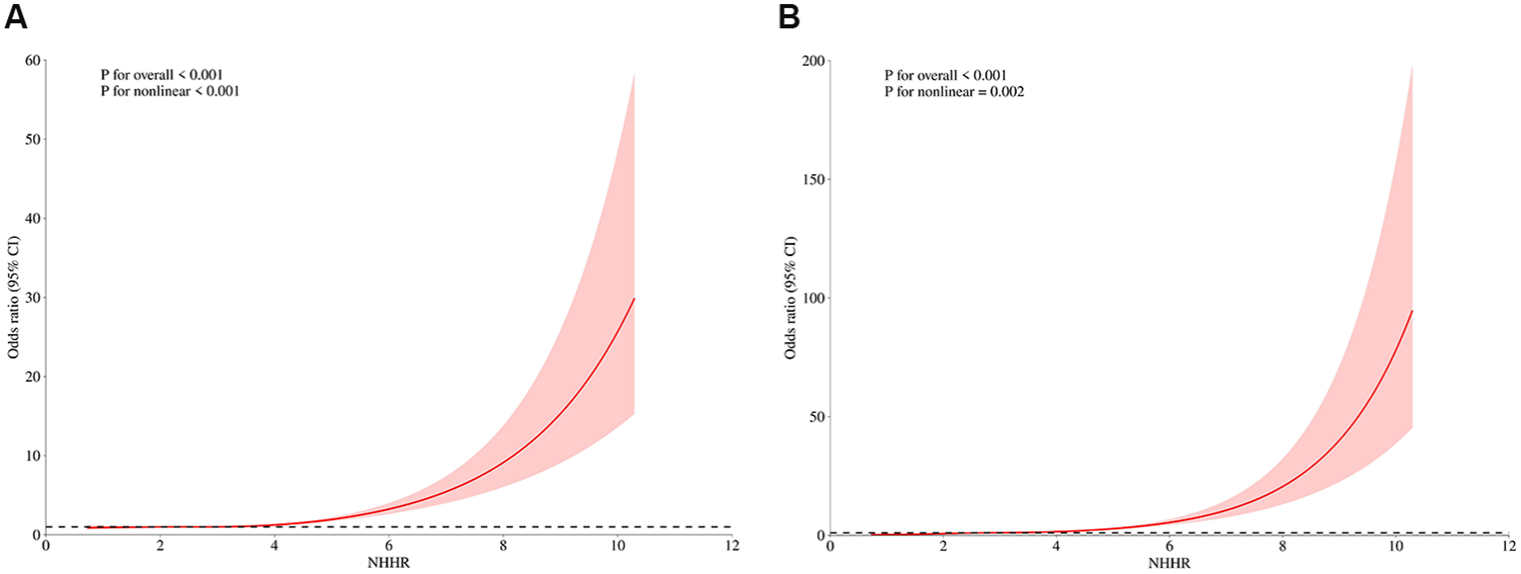
Restricted cubic splines were utilized to evaluate the hypothesis of potential nonlinear relationships between NHHR and the risk of developing new-onset hypertension (**A**) and heart diseases (**B**)

In the follow-up surveys conducted from 2013 to 2020, 1,631 participants (30.5%) were diagnosed with hypertension. The event ratio was highest in the High-NHHR group (34.7%), followed by the Medium-NHHR group (29.4%), and the Low-NHHR group (27.1%) (Table 2). The NHHR shows a positive association with the prevalence of hypertension, and this significant relationship is consistent across unadjusted, partially adjusted, and fully adjusted logistic regression models. The risk of developing hypertension varies among individuals in different NHHR groups. In the unadjusted model, the highest risk is observed in the High-NHHR group (OR = 1.723, 95% CI: 1.561–1.903, *P* < 0.001), followed by the Medium-NHHR (OR = 1.491, 95% CI: 1.087–2.043, *P* < 0.001) and Low-NHHR groups (OR = 0.935, 95% CI: 0.674–1.296, *P* < 0.001) (Table 2). Similarly, in the partially adjusted and fully adjusted models, the risk of developing new-onset hypertension among individuals in different NHHR groups follows the same trend as above. The RCS showed a partial U-shaped relationship between NHHR and incident hypertension (P for nonlinear < 0.001), indicating OR of hypertension extensively increased with increasing NHHR (Fig. 1A).

A total of 1,233 participants (23.1%) were diagnosed with new-onset heart diseases during the follow-up period from 2013 to 2020. Similar to new-onset hypertension, the incidence rate increased as NHHR grouping rose, with the highest in the High-NHHR group (34.2%), followed by the Medium-NHHR group (23.2%), and the Low-NHHR group (13.5%) (Table 3). In the unadjusted model, the highest risk was observed in the High-NHHR group (OR = 1.881, 95% CI: 1.695–2.086, *P* < 0.001), followed by the Medium-NHHR group (OR = 1.405, 95% CI: 1.000–1.973, *P* < 0.05), and the Low-NHHR group (OR = 1.069, 95% CI: 0.763–1.296, *P* < 0.05) (Table 3). The results remained consistent after adjusting for covariates. The restricted cubic spline (RCS) analysis revealed a partial U-shaped association between NHHR and the incidence of heart diseases (P for nonlinearity < 0.001), suggesting that the odds ratio (OR) for diseases significantly increased as NHHR levels rose (Fig. 1B). This result is similar to the finding for new-onset hypertension. As NHHR values increased, particularly when they exceed 8, the confidence intervals become significantly wider. This indicates greater uncertainty in the estimated risk of heart diseases at higher NHHR levels. The wider intervals may be due to a smaller sample size associated with higher NHHR values, leading to less stable estimates in those regions of the model.

**Table 2:** Association of the NHHR and the risk of developing new-onset hypertension

NHHR group	Event/total (%)	Model 1 (OR and 95% CI)	Model 2 (OR and 95% CI)	Model 3 (OR and 95% CI)
Overall	1631/5349(30.5)	1.263(1.209–1.319)	1.270(1.215–1.326)	1.265(1.210–1.323)
Low-NHHR Medium-NHHR	440/1622(27.1) 571/1941(29.4)	0.935(0.674–1.296) 1.491(1.087–2.043)	0.925(0.665–1.287) 1.482(1.080–2.033)	0.926(0.659–1.303) 1.425(1.035–1.961)
High-NHHR	620/1279(34.7)	1.723(1.561–1.903)	1.731(1.567–1.912)	1.732(1.567–1.915)

OR: odds ratio, 95% CI: 95% confidence interval

Model 1: unadjusted model

Model 2: adjusted for age, gender

Model 3: adjusted for age, gender, and marriage, education level, Hemoglobin, Creatinine, Smoked, Alcohol drink, Liver diseases, Lung diseases, Kidney diseases

**Table 3:** Association of the NHHR and the risk of developing new-onset heart diseases

NHHR group	Event/total (%)	Model 1 (OR and 95% CI)	Model 2 (OR and 95% CI)	Model 3 (OR and 95% CI)
Overall	1233/5349(23.1)	1.651(1.569–1.737)	1.654(1.572–1.740)	1.663(1.578–1.752)
Low-NHHR Medium-NHHR	172/1622(13.5) 451/1941(23.2)	1.069(0.763–1.722) 1.405(1.000-1.973)	1.119(0.692–1.811) 1.414(1.005–1.989)	1.133(0.694–1.850) 1.407(0.997–1.985)
High-NHHR	610/1786(34.2)	1.881(1.695–2.086)	1.888(1.701–2.095)	1.892(1.704–2.101)

OR: odds ratio, 95% CI: 95% confidence interval

Model 1: unadjusted model

Model 2: adjusted for age, gender

Model 3: adjusted for age, gender, and marriage, education level, Hemoglobin, Creatinine, Smoked, Alcohol drink, Liver diseases, Lung diseases, Kidney diseases

**Fig. 2:**
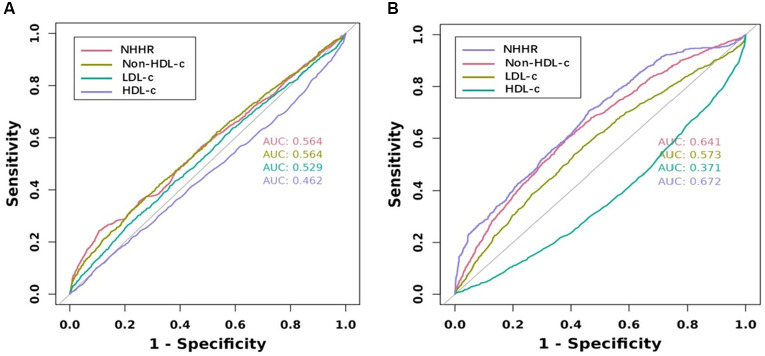
Receiver Operating Characteristics curve were utilized to evaluate the model performance of NHHR, Non-HDL-c, LDL-c and HDL-c to new-onset hypertension (**A**) and heart diseases (**B**)

**Fig. 3:**
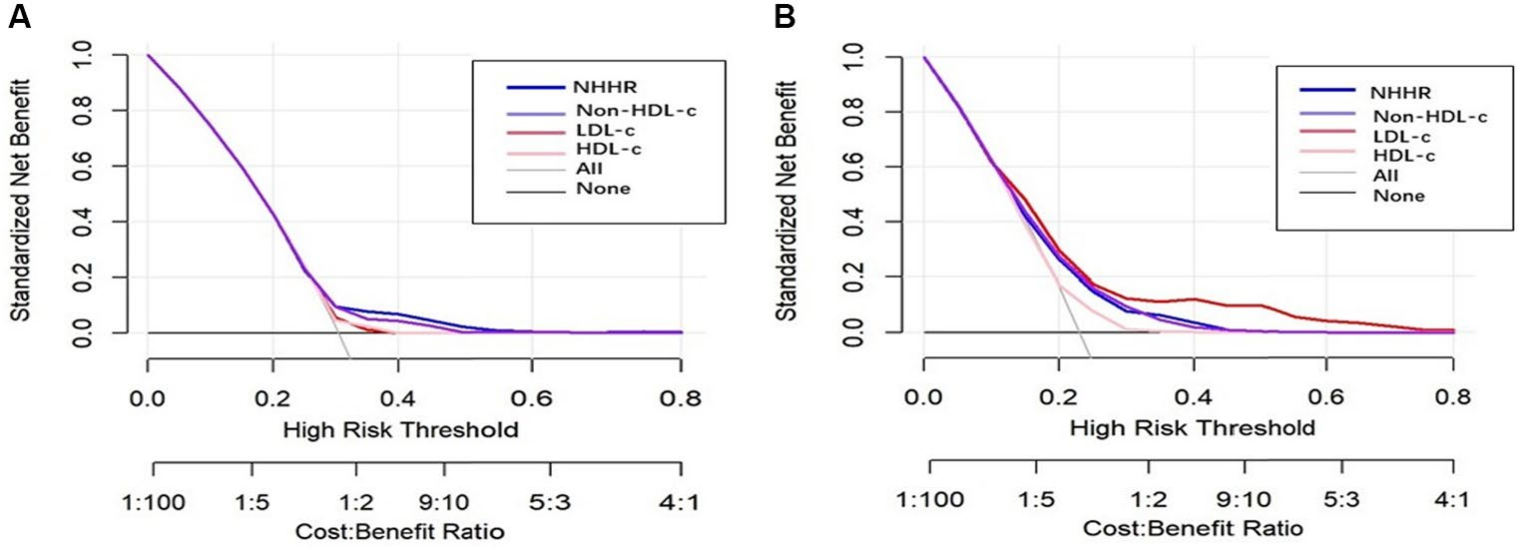
Decision curve analyses were utilized to evaluate the model performance of NHHR, Non-HDL-c, LDL-c and HDL-c to new-onset hypertension (**A**) and heart diseases (**B**)

NHHR is a novel lipid indicator that has demonstrated to have superior predictive value for diseases compared to traditional lipid markers. In this study, we also compared NHHR with LDL-C, HDL-c, and Non-HDL-c in terms of the risk of new-onset hypertension and heart diseases. In the unadjusted model, an increase in NHHR was associated with the highest risk of new-onset heart diseases (OR = 1.651, 95% CI: 1.569–1.737, *P* < 0.001) and hypertension (OR = 1.263, 95% CI: 1.209–1.319, *P* < 0.001), followed by Non-HDL-c and LDL-c (Tables 4 and 5). HDL-c has also been shown to be a significant protective indicator (OR = 0.991, 95% CI: 0.988–0.995, *P* < 0.001) and (OR = 0.968, 95% CI: 0.963–0.972, *P* < 0.001). Similarly, the partially adjusted and fully adjusted models also exhibited the same trend as mentioned above. According to the ROC curve analysis of new-onset heart diseases, the area under the curve (AUC) values for NHHR, Non-HDL-c, HDL-c and LDL-c were 56.4%, 56.4%, 46.2%, and 52.9%, respectively. For heart diseases the area under the curve (AUC) values for NHHR, Non-HDL-c, HDL-c and LDL-c were 67.2%, 64.1%, 37.1%, and 57.3%, respectively (Fig. 2). Additionally, decision curve analysis (DCA) was constructed to evaluate the clinical utility of the models. Based on our data, NHHR provided the greatest net benefit for predicting new-onset hypertension, followed by Non-HDL-c, LDL-c, and HDL-c. However, for predicting new-onset heart diseases, the net benefit was highest for LDL-c, followed by NHHR, Non-HDL-c, and HDL-c (Fig. 3).

**Table 4:** Comparison of the risks of developing new-onset hypertension

NHHR group	Model 1 (OR and 95% CI)	Model 2 (OR and 95% CI)	Model 3 (OR and 95% CI)
HDL-c	0.991(0.988–0.995)	0.990(0.987–0.994)	0.990(0.986–0.994)
LDL-c Non-HDL-c	1.003(1.001–1.005) 1.007(1.006–1.009)	1.003(1.001–1.004) 1.007(1.006–1.009)	1.006(1.004–1.008) 1.007(1.005–1.008)
NHHR	1.263(1.209–1.319)	1.270(1.215–1.326)	1.265(1.219–1.323)

OR: odds ratio, 95% CI: 95% confidence interval

Model 1: unadjusted model

Model 2: adjusted for age, gender

Model 3: adjusted for age, gender, and marriage, education level, Hemoglobin, Creatinine, Smoked, Alcohol drink, Liver diseases, Lung diseases, Kidney diseases

**Table 5:** Comparison of the risks of developing new-onset heart diseases

NHHR group	Model 1 (OR and 95% CI)	Model 2 (OR and 95% CI)	Model 3 (OR and 95% CI)
HDL-c	0.968(0.963–0.972)	0.967(0.962–0.972)	0.967(0.962–0.971)
LDL-c Non-HDL-c	1.006(1.005–1.008) 1.013(1.011–1.015)	1.001(0.994–1.008) 1.013(1.011–1.015)	1.002(1.001–1.004) 1.013(1.011–1.015)
NHHR	1.651(1.569–1.737)	1.654(1.572–1.740)	1.663(1.578–1.752)

OR: odds ratio, 95% CI: 95% confidence interval

Model 1: unadjusted model

Model 2: adjusted for age, gender

Model 3: adjusted for age, gender, and marriage, education level, Hemoglobin, Creatinine, Smoked, Alcohol drink, Liver diseases, Lung diseases, Kidney diseases

### Mediator analysis

**Fig. 4:**
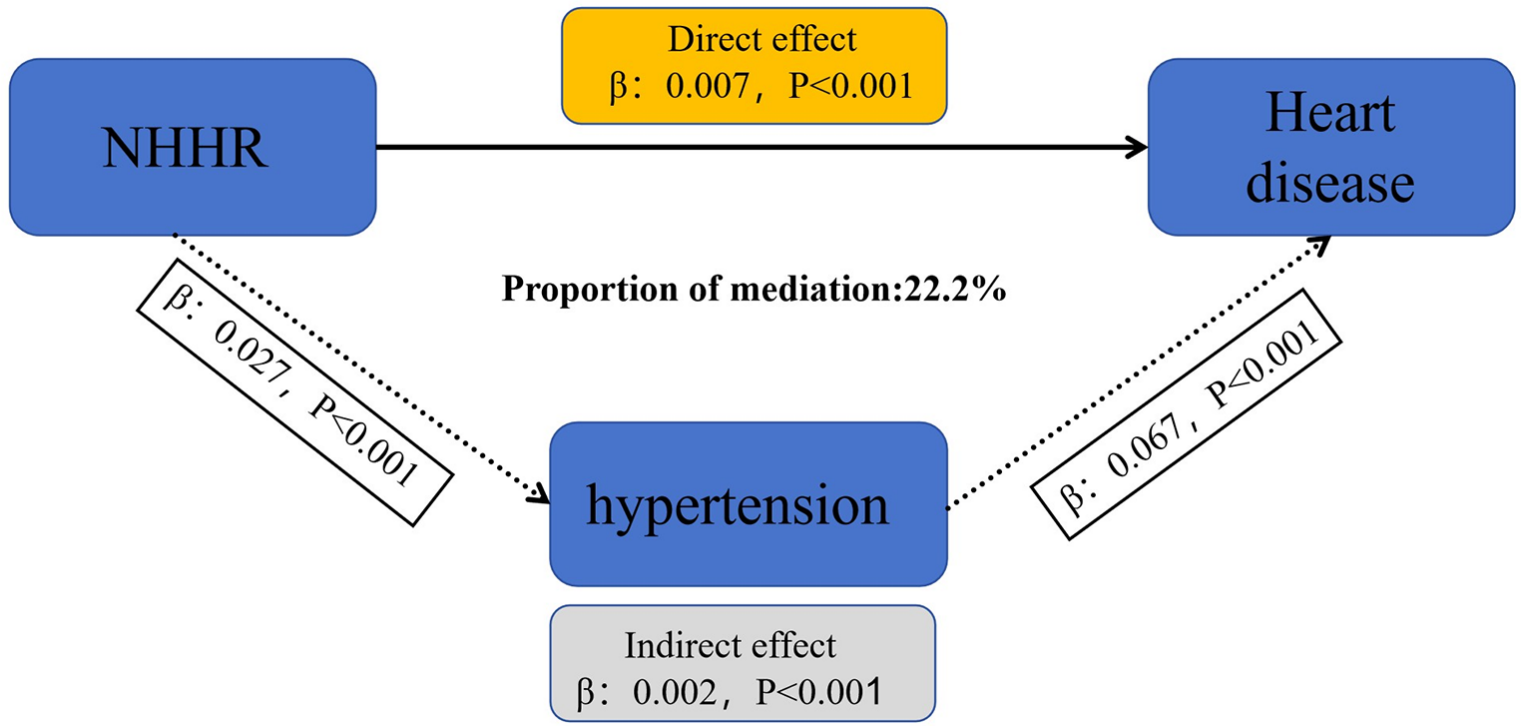
Mediation analysis of the association between the NHHR and heart diseases

Once the significant relationships between NHHR and new-onset hypertension and heart diseases were established, the mediation effect of hypertension on the link between NHHR and heart diseases was assessed using the bootstrap method [21]. The results showed that hypertension significantly influenced the relationship between NHHR and new-onset heart diseases, mediating 22.2% of the association (Fig. 4, Supplementary Table).

## Discussion

In this large-scale, nationally representative longitudinal study, we investigated the relationship between NHHR and the risk of developing hypertension and heart diseases. Our findings indicate that a higher NHHR was associated with a significantly higher odds ratio (OR) of developing these conditions compared to traditional lipid markers. Additionally, as NHHR increased, the risk of developing hypertension and heart diseases also increased. Hypertension was found to partially mediate the relationship between NHHR levels and the occurrence of heart diseases.

The NHHR, a novel lipid ratio indicator, can be easily calculated using standard lipid panel results, unlike more complex markers, making it a practical tool for widespread clinical use. NHHR incorporates both atherogenic (Non-HDL-c) and protective (HDL-c) cholesterol components, providing a more comprehensive view of lipid metabolism compared to just using LDL-C or HDL-C alone [22]. This makes it a more robust marker for heart diseases risk. Recent research has demonstrated its significant value in predicting clinical outcomes across various diseases. Liu et al., in a longitudinal cohort study of 2,253 patients with coronary artery diseases (CAD) who underwent percutaneous coronary intervention (PCI), demonstrated a U-shaped relationship between NHHR and major adverse cardiovascular and cerebrovascular events (MACCE), which aligns with the findings from the present study [23]. A cross-sectional study by Pan et al. based on the U.S. population, revealed a “J”-shaped correlation between NHHR and the risk of diabetic kidney diseases (DKD) (P for nonlinearity = 0.0136), with the lowest risk of DKD occurring when NHHR was at 2.66. The association between NHHR and DKD persisted in participants younger than 40 years, females, non-smokers, and individuals without hyperuricemia [24]. Besides, Li and colleagues found that higher levels of NHHR were associated with an increased prevalence of COPD. Mediation analysis indicated that this relationship was mediated by the dietary inflammation index, suggesting that an anti-inflammatory diet may be beneficial in reducing COPD risk [25]. These studies indirectly validate of the findings in our research, reinforcing the conclusions drawn from our analysis. Additionally, other studies have also revealed a connection between NHHR and conditions such as post-stroke depression and sleep disorders in adults, highlighting a potential link between lipid metabolism, mental health, and sleep quality [26–28].

Dyslipidemia is significantly associated with an increased risk of hypertension and heart diseases. Recent studies have offered deeper insights into this relationship. Dyslipidemia is a key factor promoting atherosclerosis, which serves as a pathological basis for both hypertension and heart diseases. For example, elevated levels of low-density lipoprotein cholesterol (LDL-c) accelerate the progression of atherosclerosis, leading to increased lipid deposition within the arteries, damaging the endothelium, and causing narrowing and stiffening of the vessels [29–30]. These structural changes in blood vessels increase vascular resistance, resulting in hypertension. Additionally, the rupture of atherosclerotic plaques can cause thrombosis, triggering acute coronary syndromes, such as myocardial infarction. Furthermore, elevated blood pressure inevitably increases the workload of the heart. If left uncontrolled, this often results in cardiac hypertrophy and remodeling, impairing the heart’s ability to relax and fill, which leads to a decline in cardiac function. Dyslipidemia also indirectly affects the risk of developing hypertension and heart diseases through various mechanisms. For example, abnormal lipid levels can trigger chronic inflammation and oxidative stress, which are major contributing factors to hypertension and cardiovascular diseases [31–32]. Research on the relationship between the Dietary Inflammation Index (DII) and NHHR suggests that an anti-inflammatory diet may help reduce NHHR levels, thereby lowering the risk of cardiovascular diseases [25]. This suggests that, in addition to lipid management, anti-inflammatory strategies also have important preventive value. Many patients with hypertension and heart diseases, or those in the early stages of these conditions, often exhibit elevated levels of non-high-density lipoprotein cholesterol (Non-HDL-c), including increased low-density lipoprotein cholesterol (LDL-c) and other components. This contributes to the development of atherosclerosis, making Non-HDL-c an excellent predictor of heart diseases [33–35]. Conversely, high-density lipoprotein cholesterol (HDL-c) has anti-inflammatory, antioxidant, and anti-atherosclerotic properties, and its levels are negatively correlated with the cardiovascular risk [36]. NHHR incorporates all lipid-related information associated with both atherogenic and anti-atherogenic processes, providing a more comprehensive balance of these factors. Higher NHHR values indicate an imbalance favoring atherogenic lipids, which can promote endothelial dysfunction, inflammation, and oxidative stress, all of which are crucial pathways in the development of hypertension and cardiovascular diseases. NHHR serves as a better indicator of the overall lipid health status of patients.

Our study divided the population into three groups according to NHHR tertiles. A baseline comparison revealed that individuals in the higher NHHR group had higher pulse rates, waist circumference, HbA1c, FBG, TG, TC, and LDL-c levels, along with more clinical comorbidities. These findings are consistent with those of Yu et al. [37], suggesting that NHHR may be an important risk factor for various conditions, such as heart diseases and diabetes. Additionally, sociodemographic comparisons revealed some interesting phenomena. In the higher NHHR group, the proportion of married individuals was relatively higher (approximately 2.93% more compared to the lowest group), whereas the proportion of individuals consuming alcohol was lower (approximately 7.27% less compared to the lowest group). Based on our findings and previous research, the corresponding diseases risk was also elevated. We speculate that these results may be related to increased life stress associated with marriage, and that moderate alcohol consumption might have a beneficial effect on lipid metabolism disorders, consistent with the results of previous studies [38–39].

The present study also demonstrated that NHHR is a significant predictor of new-onset hypertension and heart diseases. In both unadjusted and adjusted models, individuals in higher NHHR groups exhibited a markedly higher risk compared to those in the lower NHHR groups. Additionally, further RCS analysis indicated a nonlinear, partially U-shaped association between the NHHR and the risk of developing hypertension and heart diseases, consistent with previous studies on the connection between the NHHR and suicidal ideation [40]. This suggests that NHHR may serve as a valuable early indicator for identifying individuals at high risk of cardiovascular events, enabling for timely intervention. To better reflect the predictive ability of NHHR for the risk of developing hypertension and heart diseases, we compared it with common risk factors such as LDL-c, HDL-c, and Non-HDL-c. Interestingly, NHHR appears to be more effective in predicting incidence of hypertension and heart diseases, which have comparatively OR scores. This could be attributed to the fact that NHHR integrates both harmful and protective components of lipid metabolism, thus providing a more nuanced picture of an individual’s lipid health. Additionally, by constructing and comparing ROC and DCA curves, we found that NHHR has a significant advantage over other indicators in predicting the risk of heart diseases. However, in predicting hypertension risk, it ranks second to LDL-c. Studies, including those by Jiang et al. and Tan et al., have consistently shown the prognostic value of NHHR in predicting cardiovascular events and metabolic diseases, further supporting its clinical utility [41–42]. In addition, mediation analysis of our study also showed that hypertension partially mediated the association between NHHR and subsequent heart diseases. This means that the impact of NHHR on heart diseases is partially mediated by hypertension, which may be related to the accelerated damage to blood vessels and the myocardium caused by elevated blood pressure. Therefore, in the prevention and treatment of heart diseases, managing both lipid levels and blood pressure is crucial.

## Strengths and limitations of the study

The study used data from the China Health and Retirement Longitudinal Study (CHARLS), a nationally representative dataset with a large sample size and long follow-up period, providing high external validity for the findings. Multiple models were used in the study to adjust for potential confounding factors such as demographic characteristics, lifestyle, and biomarkers. By constructing ROC and DCA curves, the study compared the performance of NHHR with traditional lipid indicators in predicting the risk of hypertension and heart diseases. Furthermore, the study explored the mediating role of hypertension in the relationship between NHHR and the risk of heart diseases, revealing the potential mechanism by which NHHR increases heart diseases risk through the onset of hypertension.

Nevertheless, several limitations should be noted. Firstly, this study is based on observational data analysis, meaning that while a significant association was found between NHHR and hypertension as well as heart diseases, causality cannot be definitively established. Uncontrolled confounding factors may influence the results, preventing us from ruling out the potential impact of other variables on these associations. For example, information regarding participants’ medication use was not included in the study. Secondly, some variables in the study (such as diagnoses of hypertension and heart diseases, as well as lifestyle factors) relied on participants’ self-reports, which may lead to recall bias and reporting bias. The outcome indicators of this study are all from the self-report of the questionnaire, and some outcomes are inevitably inaccurate, which will affect the our results. In future research, we will adopt stricter diagnostic criteria or consider biomarkers that may more accurately reflect blood pressure status. Thirdly, the study used data from the China Health and Retirement Longitudinal Study (CHARLS), which mainly includes middle-aged and elderly individuals aged 45 and above, and therefore, the results may not be applicable to younger populations or other specific groups. Additionally, NHHR may change over time, particularly in long-term follow-up studies, In our paper, we primarily focused on the association between NHHR, hypertension, and cardiovascular disease. Due to the cross-sectional nature of the available data and the limited availability of time-series data, we were unable to fully explore the changes in NHHR at different time points. Future research should take this into account, particularly within the framework of longitudinal follow-up, to further evaluate the dynamic changes in NHHR and their relationship with cardiovascular events. Lastly, the uneven distribution of NHHR values in the population may reduce the model’s stability in some intervals. For example, in the RCS analysis, the small number of individuals with high NHHR values resulted in a wide confidence interval, reducing the reliability of the estimates.

## Conclusion

In a nationally representative study conducted among Chinese adults aged 45 and older, NHHR was closely associated with an increased risk of developing hypertension and heart diseases. Additionally it mediated the development of heart diseases by promoting hypertension progression. Further studies on improving NHHR could be beneficial for preventing hypertension and heart diseases.

## Electronic supplementary material

Below is the link to the electronic supplementary material.

Supplementary Material 1

Supplementary Material 2

## Data Availability

All baseline and follow-up data from participants supporting our conclusions can be found on the China Health and Retirement Longitudinal Study (CHARLS) website(https://charls.pku.edu.cn/).
