# Supplementary material for: Association of non-highdensity lipoprotein cholesterol to highdensity lipoprotein cholesterol ratio (NHHR) and subsequent hypertension and heart diseases: findings from the CHARLS cohort
Source: Aging Clin Exp Res. 2025 Jan 21;37(1):26. doi: 10.1007/s40520-024-02919-z (PMC11753329; doi:10.1007/s40520-024-02919-z)
Supplement: Supplementary file 1 — Supplementary Material 1 [file 40520_2024_2919_MOESM1_ESM.docx]

Supplementary Table Analysis of mediation

| Tests of mediation | | | | | | | | | |
| --- | --- | --- | --- | --- | --- | --- | --- | --- | --- |
| item | symbol | significance | Effect | 95% CI | | SE | *z* /*t* | *p* | Conclusion |
|  |  |  |  | Lower limit | Higher limit |  |  |  |  |
| NHHR=>hypertension=>heart diseases | a*b | Indirect effect | 0.002 | 0.002 | 0.010 | 0.002 | 1.095 | 0.274 | Partial mediation |
| NHHR=>hypertension | a | X=>M | 0.027 | 0.018 | 0.036 | 0.005 | 5.763 | 0.000 |  |
| hypertension=>heart diseases | b | M=>Y | 0.067 | 0.042 | 0.091 | 0.013 | 5.319 | 0.000 |  |
| NHHR=>heart diseases | c' | Direct effect | 0.005 | -0.003 | 0.013 | 0.004 | 1.206 | 0.228 |  |
| NHHR=>heart diseases | c | Total effect | 0.007 | -0.001 | 0.015 | 0.004 | 1.625 | 0.104 |  |
